# High-resolution magnetic resonance imaging (HRMRI) for judging the location of paraclinoid aneurysms (PAs): assisting in diagnosis and treatment decision of PAs

**DOI:** 10.1186/s41016-025-00420-8

**Published:** 2026-01-26

**Authors:** Xiaohui Hou, Jiewen Geng, Simin Wang, Xinxin Fan, Sishi Xiang, Peng Hu, Chuan He, Mingchu Li, Hongqi Zhang

**Affiliations:** 1 Xuan Wu Hospital of the Capital Medical University, Beijing, China; 2 Second Affiliated Hospital of Xingtai Medical College, Hebei, China; 3 Xi’an NO.3 Hospital, the Affiliated Hospital of Northwest University, Shanxi, China

**Keywords:** High-resolution magnetic resonance imaging, Paraclinoid aneurysms, Diagnosis

## Abstract

**Background:**

Determining the location of paraclinoid aneurysms (PAs) is crucial. We aimed to evaluate the utility of paraclinoid high-resolution MRI (HRMRI) in determining PA locations.

**Methods:**

We enrolled patients with suspected PAs who underwent our HRMRI sequence in 6 months. PAs were categorized into five types based on their origin from the internal carotid artery (ICA): Superior ophthalmic segment (Type S), Ventral ophthalmic segment (Type V), Medial clinoidal segment (Type M), Lateral clinoidal segment Type L, and Posterior clinoidal segment) (Type P). The paraclinoid HRMRI protocol included five main sequences: TOF-MRA, coronal and saggital high-resolution T2-weighted images, coronal and saggital enhanced high-resolution T1-weighted images. We utilized cerebrospinal fluid (CSF) notch and cavernous sinus enhanced signals to determine the location of PAs.

**Results:**

Sixty-nine patients with 75 PAs were included. Based on our classification, there were 10 Type S, 2 Type V, 45 Type M, 11 Type L, and 7 Type P PAs. Among the Type S PAs, 9 were fully located within the subarachnoid space, and 1 was in the juncture area. Both Type V PAs were situated within the cavernous sinus. Among the Type M PAs, 34 were located in the cavernous sinus, and 1 was in the juncture. Of the Type L PAs, 5 were within the cavernous sinus, and 1 was in the juncture area. All 7 Type P PAs were located within the cavernous sinus.

**Conclusions:**

HRMRI sequences may assist in determining the location of PAs and could provide useful information for clinical decision-making, especially when radiation-free or iodine-free evaluation is preferred.

**Trial registration:**

The clinical trial of China Internal Aneurysm Project (NCT03115905).

## Background

Intracranial aneurysms (IAs) represent a pathological dilation of intracranial blood vessel walls. Subarachnoid hemorrhage (SAH) resulting from IA rupture is the most common cause of non-traumatic SAH, associated with extremely high mortality and disability rates [1, 2]. Unruptured aneurysms (UIAs) have a high incidence rate, affecting 3.2% to 7% of adults [3, 4], yet their annual rupture rate is only 0.25% to 2.00% [5, 6]. Physicians have long been concerned about evaluating and predicting the long-term risk of UIA rupture to guide treatment decisions.

Paraclinoid aneurysm (PA) arises from the ophthalmic segment of the internal carotid artery (ICA) extending to adjacent structures, also referred to as the Bouthillier and associates’ nomenclature C4–C6 segment [7, 8]. Previous studies have indicated that this type of aneurysm accounts for a significant proportion of all UIAs, approximately 32% to 53.9% [3, 4]. Aneurysms at this location are considered to have the lowest risk of rupture [9], as they may be proximal to the dural ring or even within the cavernous sinus, thus not causing subarachnoid hemorrhage [10]. Therefore, evaluating and predicting the rupture risk of UIAs involves determining whether the UIA is located in the paraclinoid portion and if the paraclinoid aneurysm is situated in the cavernous sinus.

Interestingly, currently published UIA risk prediction scores or models do not appear to fully incorporate this crucial criterion, potentially leading to selection bias [11]. We combined previous imaging methods, primarily HRMRI, with our modifications to develop a new protocol for determining the location of PAs, offering innovative insights into predicting the rupture risk of PAs and overall UIAs and guiding treatment decisions. Rather than claiming technical novelty, this study aims to integrate existing imaging markers into a practical workflow that may help clinicians better differentiate intra-dural from extra-dural paraclinoid aneurysms and guide individualized management strategies.

## Methods

### Study design and population

We retrospectively collected data from patients in our hospital from February 15, 2023, to August 15, 2023, who were suspected of having PAs and underwent HRMRI. This research protocol was approved by the Ethics Committee of Xuanwu Hospital, Capital Medical University (No. 2016032), and was registered for inclusion in the clinical trial of China Internal Aneurysm Project (NCT03115905). All participants provided written informed consent. The study followed the Declaration of Helsinki and the Good Clinical Practice for Medical Device Trials (Order No. 25 issued by the China Food and Drug Administration and the National Health and Family Planning Commission). Ethical approval and informed consent were obtained, and the study protocol was reviewed and approved by institutional review boards.

Inclusion criteria: (1) diagnosis of untreated PA by MRA, CTA, or DSA; (2) underwent HRMRI; (3) patient or guardian signed an informed consent form. Exclusion criteria: (1) reconstruction indicating no aneurysm or the aneurysm was located in other segments; (2) presence of non-saccular aneurysms such as dissecting, traumatic, or bacterial aneurysms; (3) inability of the patient to adhere to magnetic resonance imaging resulting in poor imaging quality.

### Classification of PAs

**Fig. 1:**
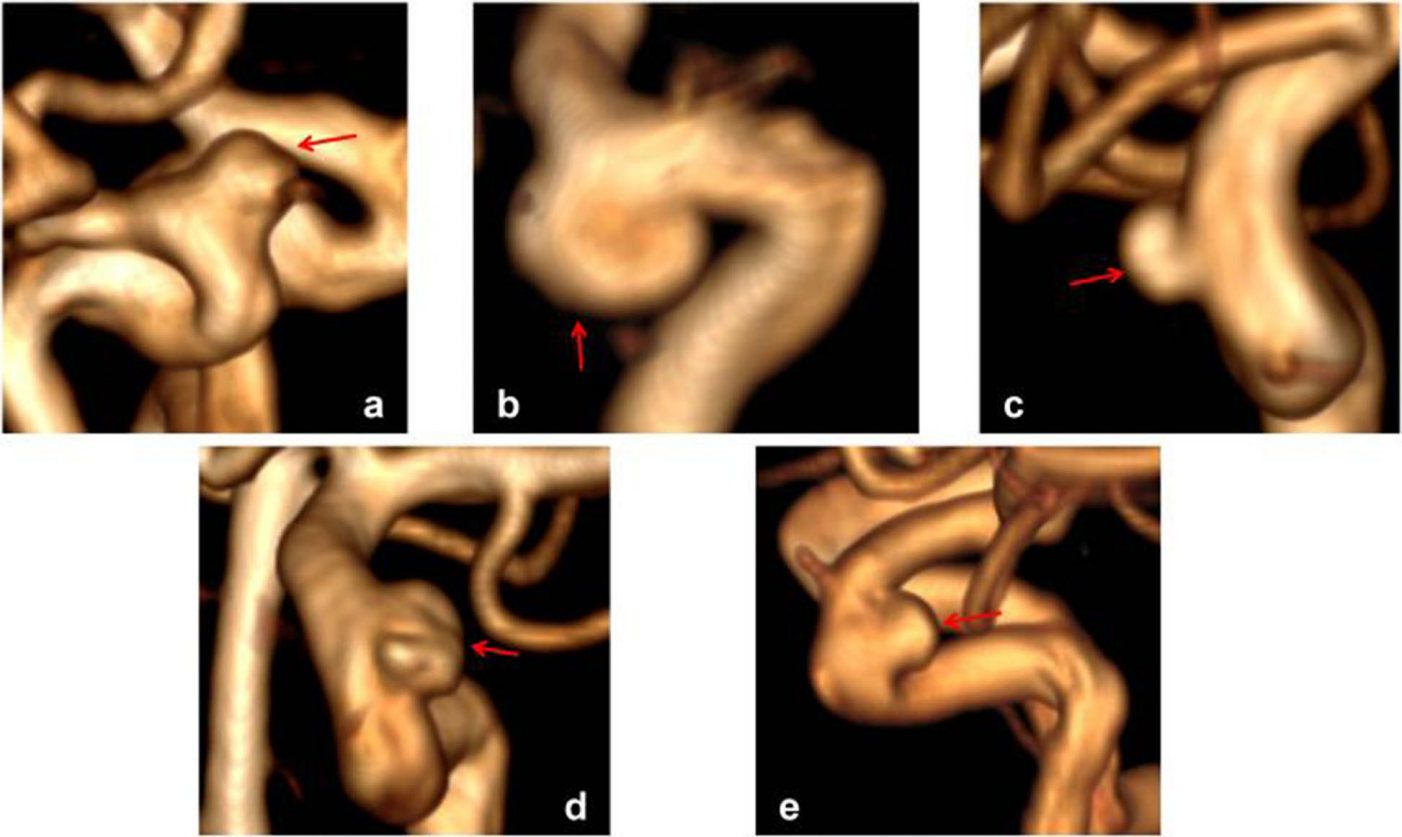
Different types of PAs

While the classification proposed by Barami in 2003 was convenient and practical [8], it does not fully include aneurysms arising laterally and located in the posterior clinoidal segment. Therefore, based on the Barami classification, we propose our classification (Fig. 1):

1. Type S: aneurysm arising from the superior ophthalmic segment of the ICA, equivalent to types Ia and Ib in the Barami classification (Fig. 1a).
2. Type V: aneurysms arising from the ventral ophthalmic segment of the ICA, equivalent to type II in the Barami classification (Fig. 1b).
3. Type M: aneurysms arising from the medial clinoidal segment of the ICA, equivalent to types IIIa and IIIb in the Barami classification (Fig. 1c).
4. Type L: aneurysms arising from the lateral clinoidal segment of the ICA (Fig. 1d).
5. Type P: aneurysms arising from the posterior clinoidal segment of the ICA, primarily in the posterior aspect of the siphon (Fig. 1f).

### MR protocol

**Fig. 2:**
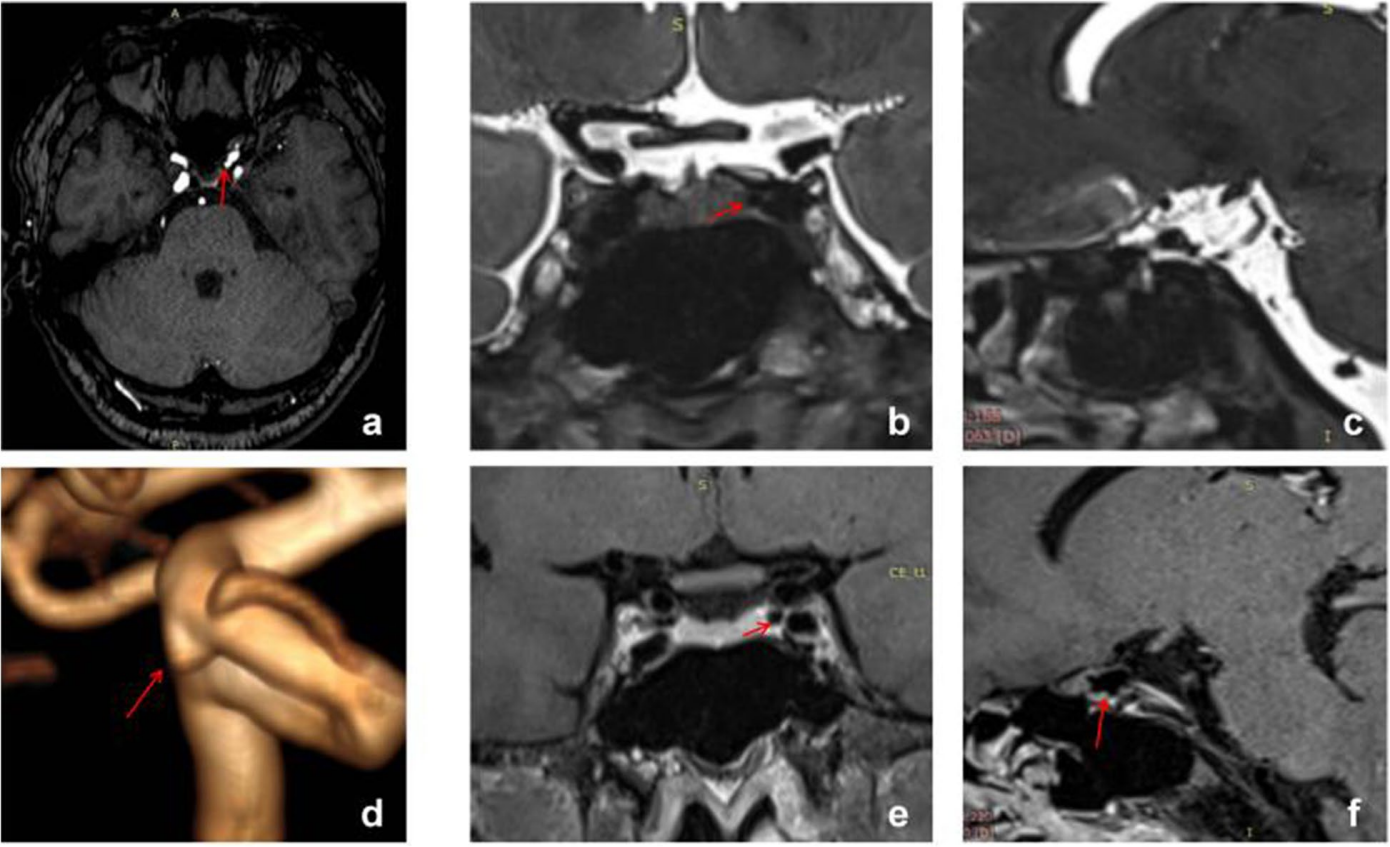
Interpretation of a Type M PA, where the red arrow denotes the location of the aneurysm. Panel **d** clearly illustrates the Type M aneurysm, while panels **e** and **f** facilitate easy determination of the aneurysm's location within the cavernous sinus enhancement signal

All MRI studies were conducted using a 3-T magnetic resonance scanner (Verio, Siemens, Erlangen, Germany) equipped with a 20-channel head coil. Drawing from previous literature, we devised a novel protocol to identify the location of PAs by integrating TOF-MRA, high-resolution T2-weighted images, and enhanced high-resolution T1-weighted images. The protocol was as follows:

1. TOF-MRA: TR, 22 ms; TE, 3.74 ms; slice thickness, 0.5 mm; slices per slab, 96; slice oversampling, 16.7%; phase oversampling, 0%; FOV, 20 cm; and voxel size, 0.3*0.3*0.5 mm. TOF-MRA primarily served for reconstruction and initial determination of aneurysm location (Fig. 2a, d).
2. Coronal high-resolution T2-weighted images: TR, 2000 ms; TE, 32 ms; slice thickness, 0.4 mm; slices per slab, 128; slice oversampling, 12.5%; phase oversampling, 0%; FOV, 18 cm; and voxel size, 0.5*0.5*0.4 mm. Coronal high-resolution T2-weighted images primarily depicted the relationship between aneurysms and the subarachnoid space, particularly suitable for aneurysms arising from the median or lateral aspect of the ICA (Fig. 2b).
3. Sagittal high-resolution T2-weighted images: TR, 2000 ms; TE, 29 ms; slice thickness, 0.4 mm; slices per slab, 144; slice oversampling, 33.3%; phase oversampling, 0%; FOV, 18 cm; and voxel size, 0.6*0.6*0.4 mm. Sagittal high-resolution T2-weighted images also depicted the relationship between aneurysms and the subarachnoid space, particularly for aneurysms arising from the superior, ventral, or posterior aspect of the ICA (Fig. 2c).
4. Coronal enhanced high-resolution T1-weighted images: TR,900 ms; TE, 18 ms; slice thickness, 0.4 mm; slices per slab, 256; slice oversampling, 50%; phase oversampling, 25%; FOV, 18 cm; and voxel size, 0.5*0.5*0.4 mm. Coronal enhanced high-resolution T1-weighted images depicted the relationship between aneurysms and the cavernous sinus, particularly for aneurysms located in the median or lateral segment of the ICA (Fig. 2e).
5. Sagittal enhanced high-resolution T1-weighted images: TR,800 ms; TE, 16 ms; slice thickness, 0.4 mm; slices per slab, 240; slice oversampling, 33.3%; phase oversampling, 0%; FOV, 18 cm; and voxel size, 0.6*0.6*0.4 mm. Sagittal enhanced high-resolution T1-weighted images were also utilized to depict the relationship between aneurysms and the cavernous sinus, particularly for those arising from the superior, ventral, or posterior aspect of the ICA (Fig. 2f).

### Image analysis

Reconstruction and interpretation were conducted using the open-source software Radiant Dicom Viewer. We primarily determined whether the aneurysm was located in the cavernous sinus or subarachnoid space based on the T2 phase cerebrospinal fluid notch and cavernous sinus enhancement signal. As depicted in Fig. 2, we employed TOF-MRA reconstruction to ascertain that it was a Type M aneurysm in the left ICA paraclinoid segment (Fig. 2d). The coronal enhanced high-resolution T1-weighted image illustrated that the aneurysm was entirely situated in the cavernous sinus (Fig. 2e), while the coronal high-resolution T2-weighted images indicated the absence of the aneurysm in the cerebrospinal fluid (Fig. 2b). Consequently, the aneurysm was determined to be located in the cavernous sinus of the PA. Additionally, it was observed that the sagittal view might be challenging to assess due to vessel occlusion (Fig. 2c, f). The interpretation was conducted by a neurosurgeon with over 5 years of experience in treating aneurysms and was reviewed by a senior doctor.

### Statistical analysis

This study primarily employed descriptive statistical analysis of the enrolled cases. All categorical variables were presented as absolute numbers with percentages, while continuous variables were assessed for normality using P-P plots. Continuous variables were reported as means ± SDs or as medians and interquartile ranges. All statistical analyses were conducted using SPSS version 26.0 (IBM, NY, USA).

## Results

### Population

According to the inclusion criteria, a total of 98 patients suspected of having paraclinoid aneurysms were initially considered. However, 8 patients were excluded after reconstruction revealed they had fundibulum rather than aneurysms. Additionally, 8 patients were excluded because their aneurysms were located in non-paraclinoid portions of the ICA. Furthermore, 13 patients were excluded due to inability to cooperate with HRMRI or poor image quality. Ultimately, 69 patients with 75 PAs were included in the analysis.

Among the 69 patients, 26 were male, and 6 patients had two paraclinoid aneurysms. Of the 75 aneurysm cases, 42 were located on the left side. According to the classification used in this study, there were 10 cases of Type S, 2 cases of Type V, 45 cases of Type M, 11 cases of Type L, and 7 cases of Type P (Table 1).

**Table 1:** Baseline of the patients and aneurysms

Patient (*n* = 69)	Characteristics (%)
Male	26 (37.7)
Multiple aneurysms	6 (8.7)
Aneurysms (*n* = 75)	
Diameter	3.9 ± 2.1
Neck width	4.5 ± 1.4
Left side	42 (56.0)
Classification	
Type S	10 (13.3)
Type V	2 (2.7)
Type M	45 (60.0)
Type L	11 (14.7)
Type P	7 (9.3)

### Interpretation result

Among the 10 Type S aneurysms, 9 were situated entirely in the subarachnoid space, while 1 was located at the juncture area. Both Type V aneurysms were found within the cavernous sinus. Of the 45 Type M aneurysms, 34 were entirely located in the cavernous sinus, 10 were situated in the subarachnoid space, and 1 was at the juncture area. Regarding the 11 Type L aneurysms, 5 were entirely within the cavernous sinus, 5 were in the subarachnoid space, and 1 was at the juncture area. All 7 Type P aneurysms were located within the cavernous sinus (Table 2).

**Table 2:** Relationship between types of aneurysm and their locations

Classification	Cvernous sinus (%)	Subarachnoid space (%)		Juncture (%)
Type S (*n* = 10)	0 (0.0)	9 (90.0)	1 (10.0)	
Type V (*n* = 2)	2 (100.0)	0 (0.0)	0 (0.0)	
Type M (*n* = 45)	34 (75.6)	10 (22.2)	1 (2.2)	
Type L (*n* = 11)	5 (45.5)	5 (45.5)	1 (9.1)	
Type P (*n* = 7)	7 (100.0)	0 (0.0)	0 (0.0)	
Total (*n* = 75)	48 (64.0)	24 (32.0)	3 (4.0)	

### Typical cases

**Fig. 3:**
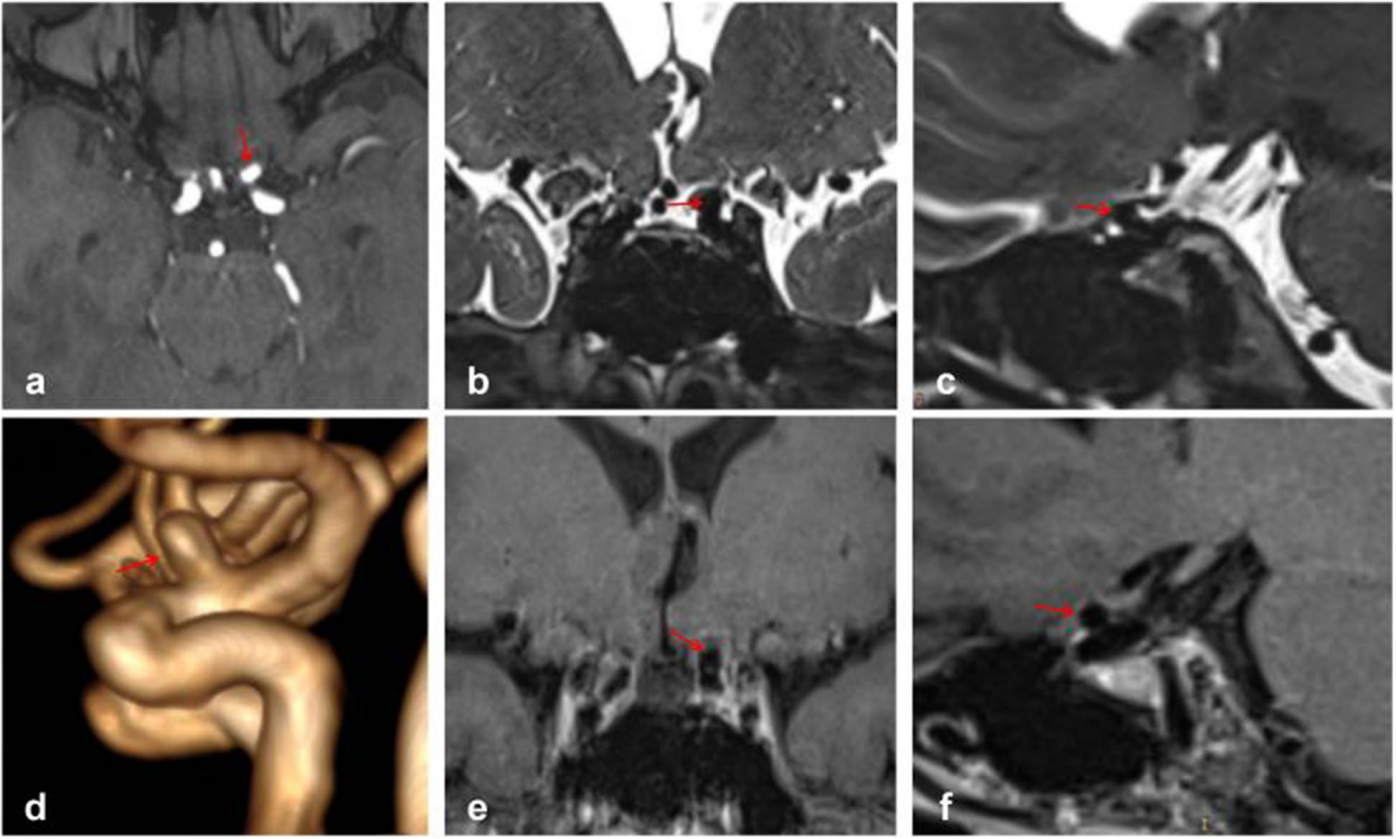
Aneurysm of Type S, where the red arrow indicates the location of the aneurysm. Panel **d** clearly depicts the Type S aneurysm, while panels **b** and **c** facilitate easy determination of the aneurysm’s location within the cerebrospinal fluid signal, indicating it is in the subarachnoid space

**Fig. 4:**
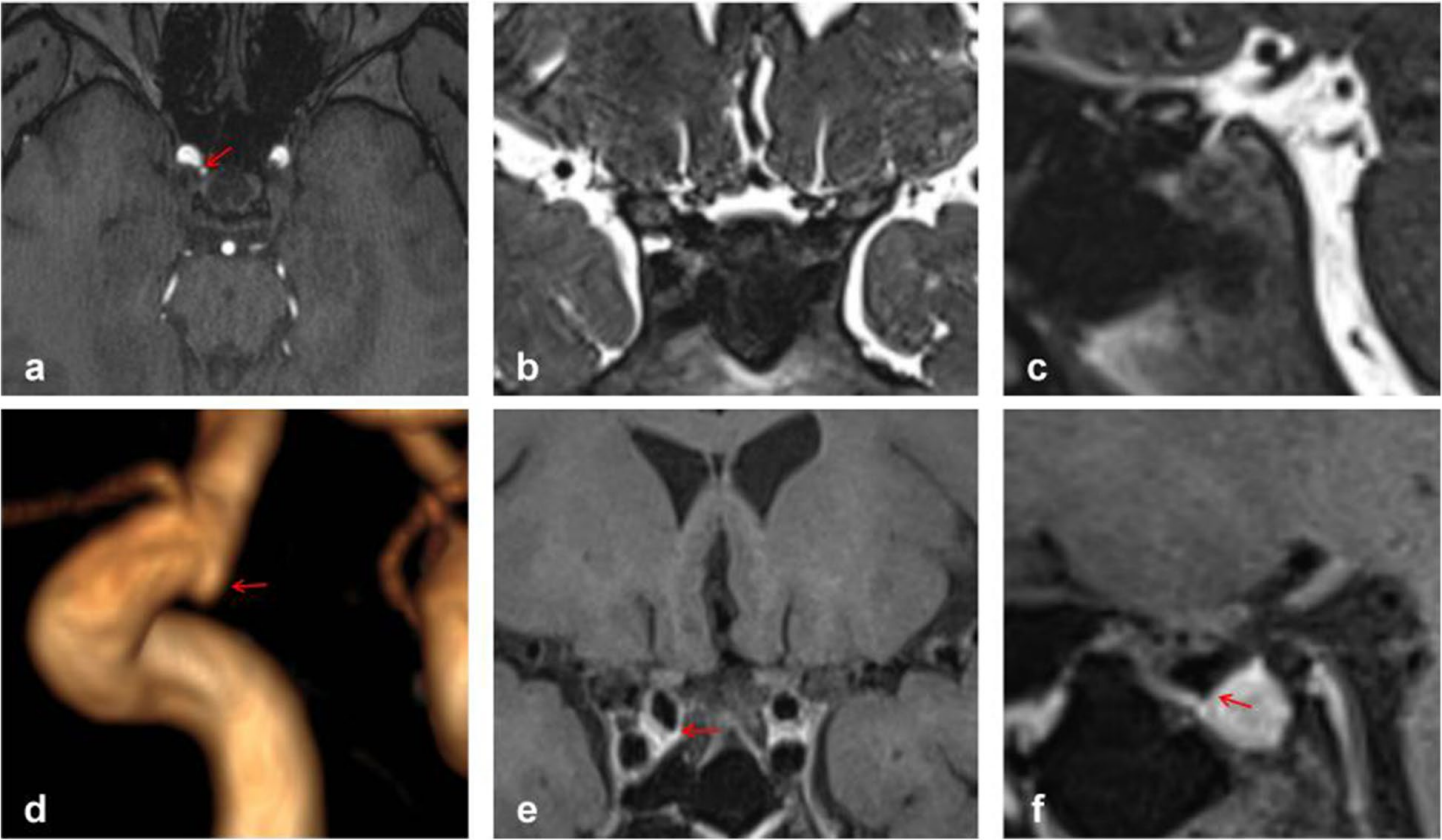
Aneurysm of Type V, where the red arrow indicates the location of the aneurysm. Panel **d** provides a clear view of the Type V aneurysm, while panels **e** and **f** enable easier determination of the aneurysm’s location within the cavernous sinus enhancement signal, confirming its location in the cavernous sinus

**Fig. 5:**
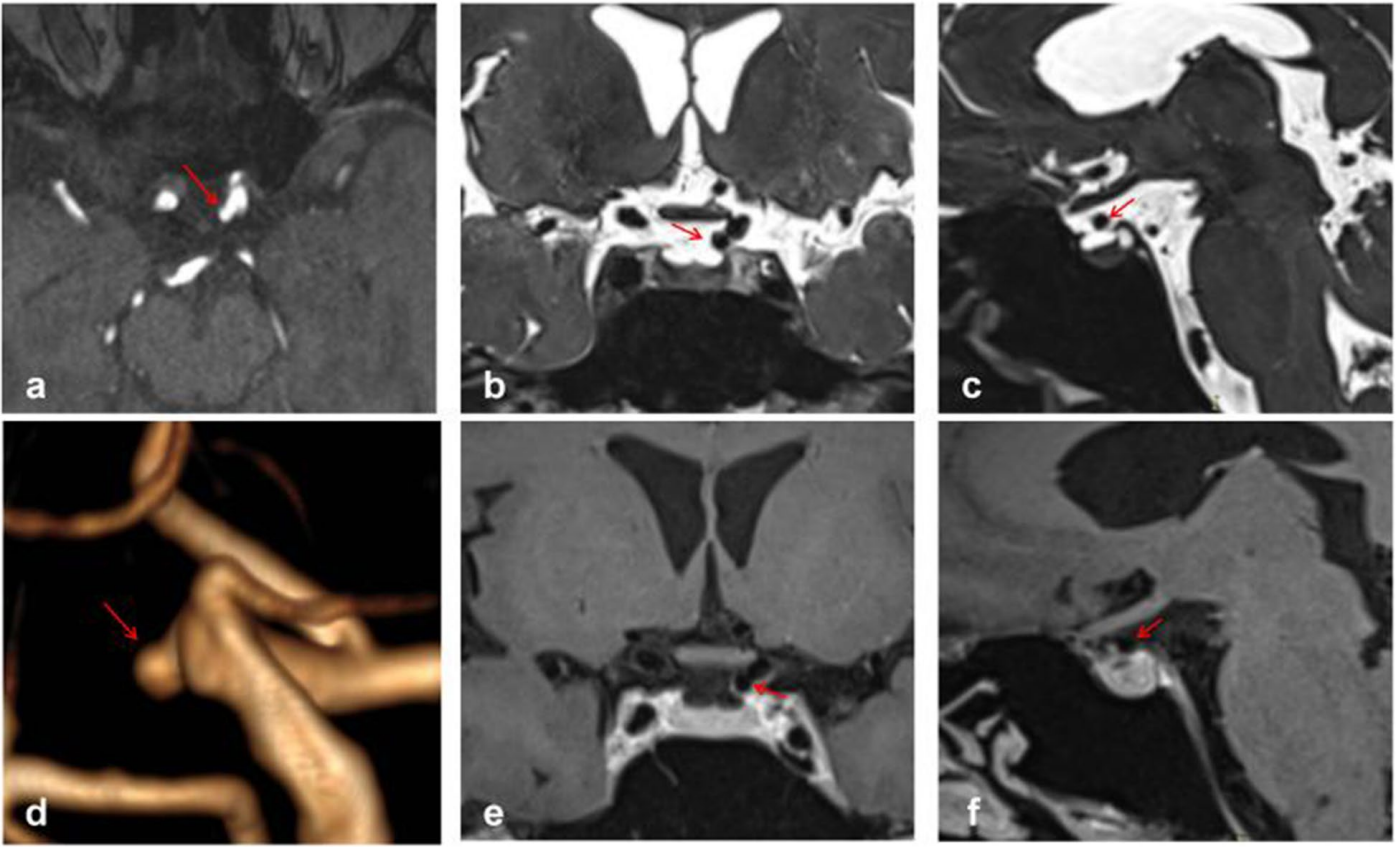
Aneurysm of Type M, where the red arrow indicates the location of the aneurysm. Panel **d** clearly shows the Type M aneurysm, while panels **b** and **c** facilitate easier determination of the aneurysm’s location within the cerebrospinal fluid, indicating its location in the subarachnoid space

**Fig. 6:**
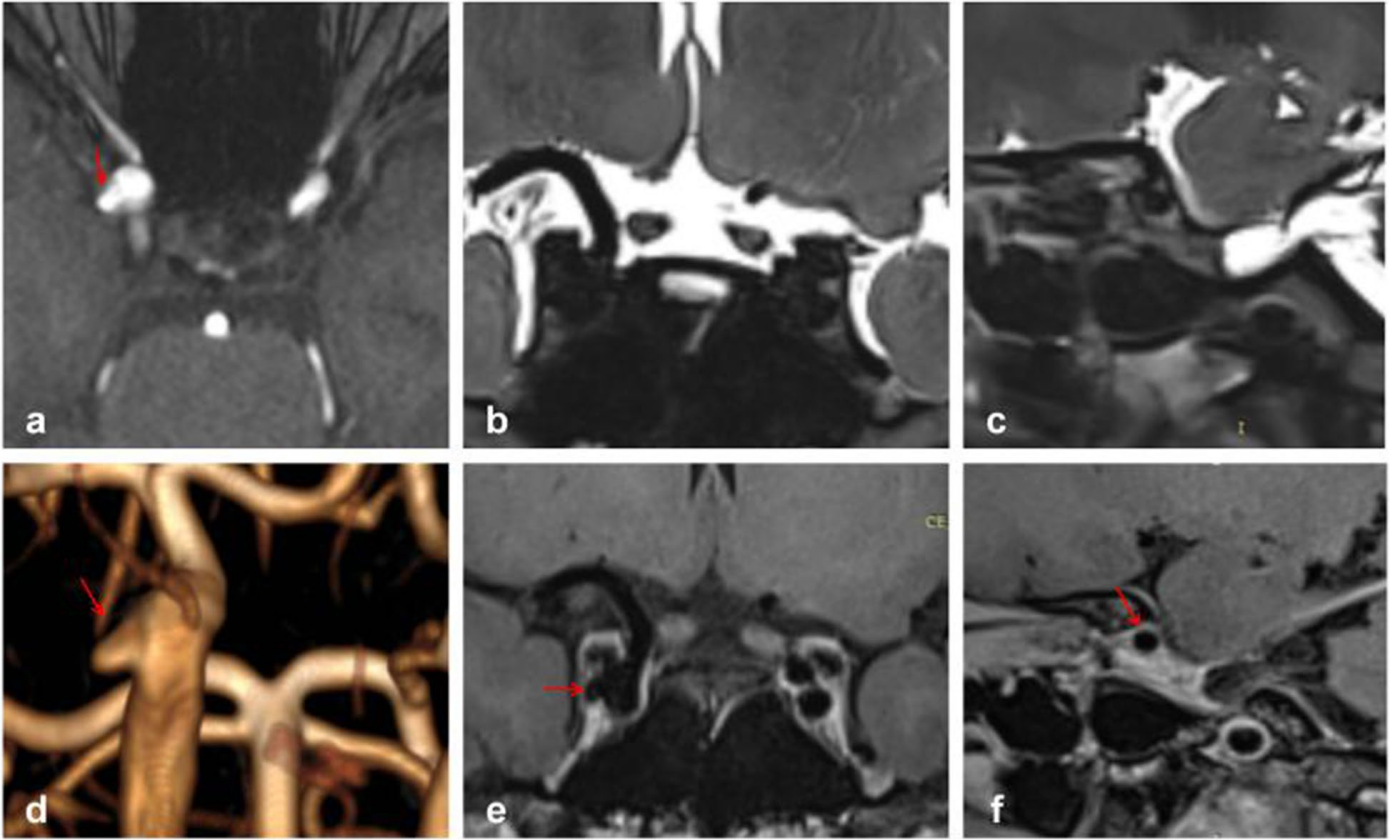
Aneurysm of Type L, where the red arrow indicates the location of the aneurysm. Panel **d** provides a clear view of the Type L aneurysm, while panel **e **enables easier determination of the aneurysm’s location within the cavernous sinus enhancement signal, confirming its location in the cavernous sinus

**Fig. 7:**
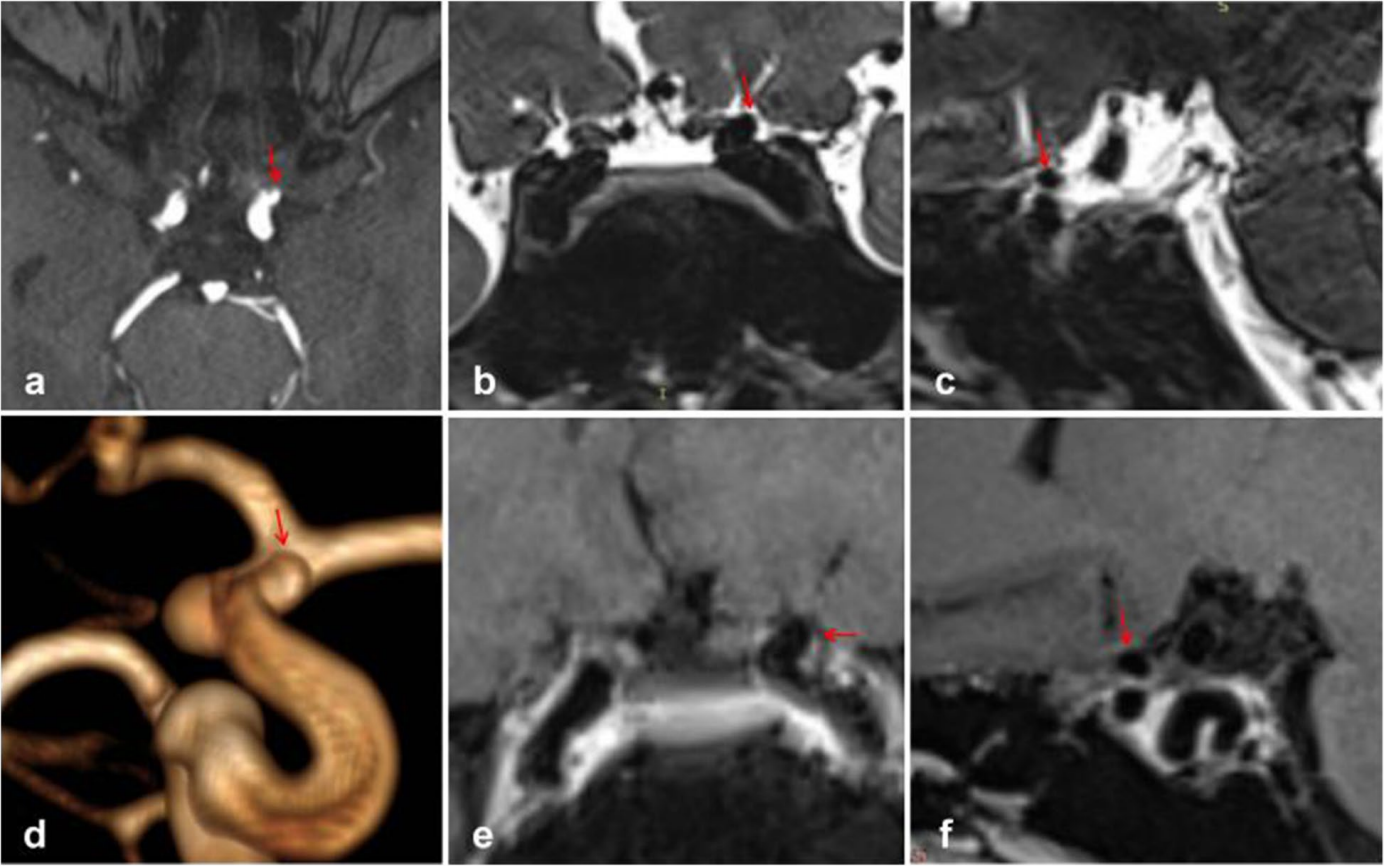
Aneurysm of Type L, where the red arrows indicate the location of the aneurysm. Panel **d** clearly depicts the Type L aneurysm, while panels **b** and **e** facilitate easier determination of the aneurysm’s location within the cerebrospinal fluid signal, indicating its location in the subarachnoid space

**Fig. 8:**
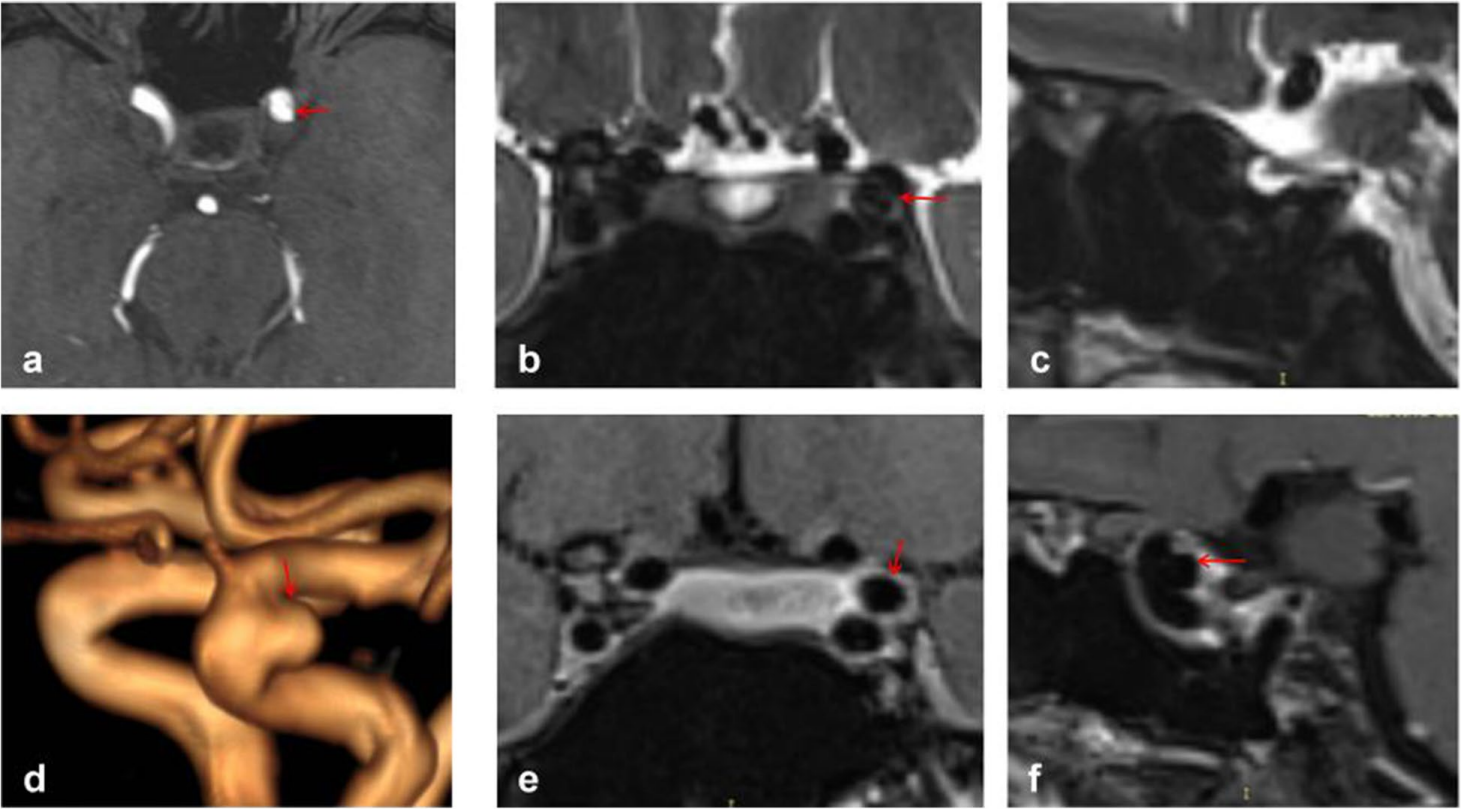
Aneurysm of Type P, where the red arrow indicates the location of the aneurysm. Panel **d **provides a clear view of the Type P aneurysm, while panels **e** and **f** enable easier determination of the aneurysm's location within the cavernous sinus enhancement signal, confirming its location in the cavernous sinus

**Fig. 9:**
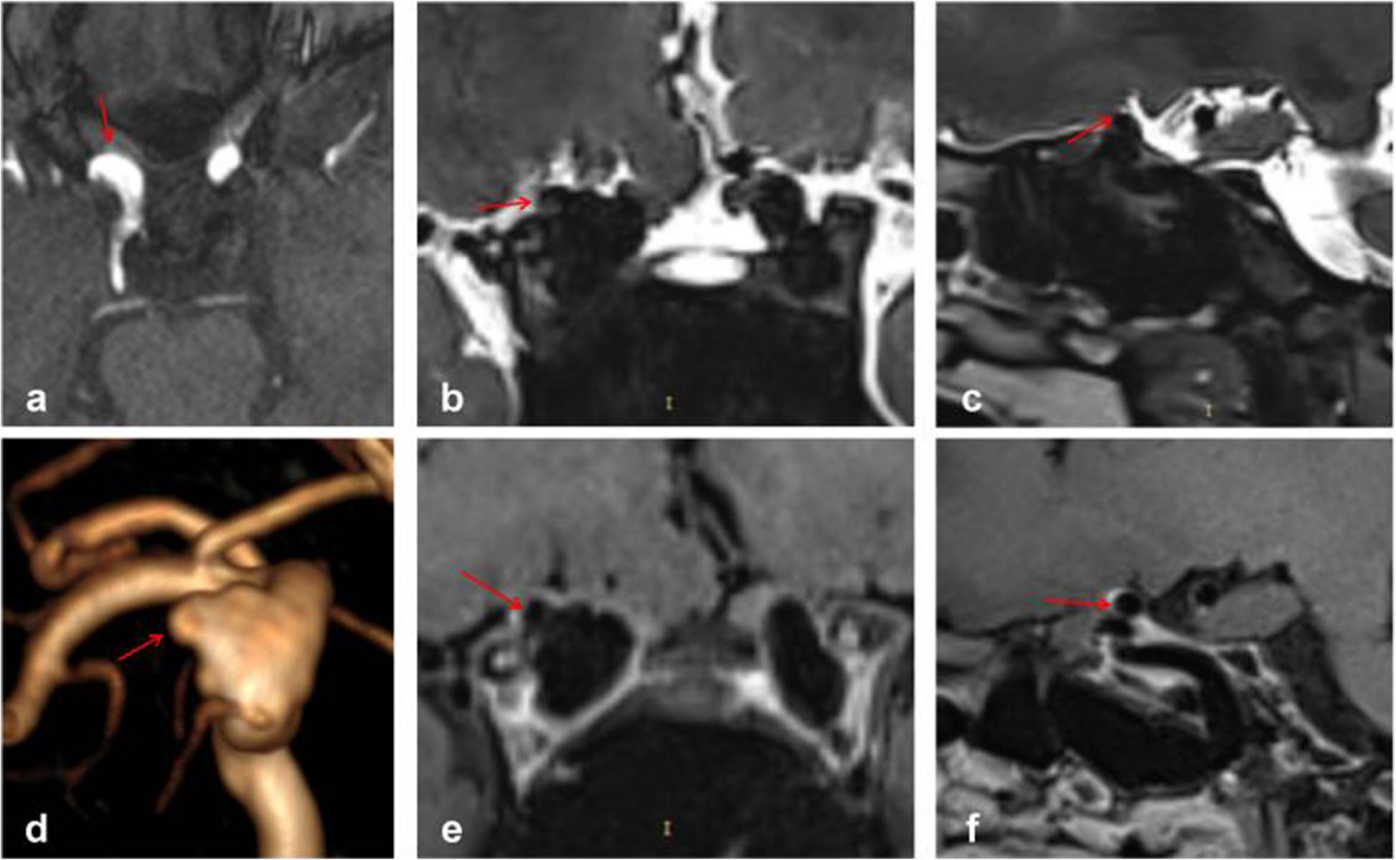
Aneurysm of Type L, where the red arrows indicate the position of the aneurysm. This panel clearly depicts the Type L aneurysm, while panels **b**, **c**, and **e** facilitate easier determination of the aneurysm’s location within the cavernous sinus enhancement signal and partly within the cerebrospinal fluid signal, indicating juncture area aneurysms

Figure 3 depicts typical cases of Type S aneurysms located in the subarachnoid space, while Fig. 4 illustrates typical cases of Type V aneurysms located in the cavernous sinus. Furthermore, Fig. 5 shows typical cases of Type M aneurysms situated in the subarachnoid space, and the typical cases of Type M aneurysms located in the cavernous sinus are presented in Fig. 2. Additionally, Fig. 6 showcases typical cases of Type L aneurysms located in the cavernous sinus, while Fig. 7 displays typical cases of Type L aneurysms situated in the subarachnoid space. Moreover, Fig. 8 exhibits typical cases of Type P aneurysms located in the cavernous sinus, and Fig. 9 presents typical juncture area aneurysms (Type L).

It is noteworthy that the diameters of the three juncture area aneurysms were 5.01 mm, 9.41 mm, and 13.90 mm, respectively. The latter two aneurysms were the largest in this group.

In addition, all aneurysms were assessed for the presence of wall enhancement on high-resolution magnetic resonance imaging (HR-MRI). The results demonstrated no evidence of wall enhancement in any of the cases.

## Discussions

There has been considerable research focusing on the relationship between PAs and the distal dural ring (DDR). Previous studies often relied on DSA to determine the location of the aneurysm, primarily assessing its relationship with the origin of the ophthalmic artery [12]. However, the variability in the origin of the ophthalmic artery among patients renders this diagnostic approach relatively unreliable [13]. Some studies attempted to determine aneurysm location through CTA, primarily relying on the upper edge of the optic strut (OS) as the anterior boundary of the DDR. While CTA facilitates observation of the relationship between blood vessels, aneurysms, and OS [14], OS only represents the upper limit of DDR and may not be applicable for diagnosing all PAs originating from different segments of the ICA. Moreover, some studies have suggested that aneurysms originating below OS can still cause subarachnoid hemorrhage, rendering this judgment method controversial [15, 16].

HRMRI has also been investigated for determining PA locations [10, 17], primarily focusing on markers such as the junction between the sellar diaphragm and the top of the cavernous sinus, the interior of the anterior clinoid process, and the cerebrospinal fluid notch. This method has been validated based on surgical findings of PAs, demonstrating a sensitivity of 92.3% and specificity of up to 100% [18]. Our research builds upon this foundation and proposes a refined MR protocol with systematic sequence combination.

Compared to CTA, which infers the position of the DDR indirectly from bony anatomy, our HRMRI protocol directly visualizes cerebrospinal fluid (CSF) flow voids and post-contrast enhancement of the cavernous sinus, offering a soft-tissue-based assessment. Although CTA excels in depicting bony relationships (e.g., with the anterior clinoid process), our approach focuses on direct visualization of anatomical compartments, potentially reducing reliance on variable osseous landmarks.

Our imaging approach includes the addition of T1-enhanced images and the integration of MRA reconstruction to pinpoint the location of PA. Simultaneously, we conduct HRMRI scans in both sagittal and coronal sections. This comprehensive approach ensures clear identification of the aneurysm regardless of its origin within the internal carotid artery or its directional orientation. Our method has been streamlined to facilitate determination of whether the aneurysm is situated in the cavernous sinus or the subarachnoid space, reducing the necessity of assessing the DDR to some extent.

Based on our observations, it can be inferred that aneurysms arising from the superior ophthalmic segment of the ICA are predominantly located in the subarachnoid space, suggesting a potential treatment need for this type of aneurysm. Aneurysms originating from the ventral ophthalmic segment and posterior clinoidal segment of the ICA are more likely to be found in the cavernous sinus and may warrant follow-up monitoring. If the patient cannot undergo prolonged HRMRI scans, preliminary judgment can be made based on the aneurysm type. Paraclinoid HRMRI proves to be particularly valuable for aneurysms pointing medially or laterally. Our findings suggest that HRMRI is highly beneficial for such cases, provided there are no contraindications to MR imaging.

However, although our imaging sequence can ascertain whether the aneurysm is located in the cavernous sinus, this does not negate the need for intervention in cavernous sinus aneurysms. Previous studies have indicated that cavernous carotid aneurysms may exhibit a propensity for enlargement due to a lack of constraint. Enlarged cavernous sinus aneurysms may lead to syndromes or even rupture through the dura [19]. Therefore, even if our sequence determines that the aneurysm is situated in the cavernous sinus and poses no immediate risk of rupture, long-term follow-up observation remains essential.

Our study indicates that many previous investigations into the rupture risk of aneurysms may be insufficient, particularly in evaluating aneurysms in the paraclinoid segment [11]. Aneurysms in the ophthalmic segment, included in prior aneurysm studies, may not have been assessed for their specific locations, potentially leading to the inclusion of some epidural aneurysms in the analysis. The rupture risk of these aneurysms is considerably lower than that of intradural aneurysms, warranting separate discussion. We advocate for distinguishing the location of aneurysms in the paraclinoid segment as a crucial step to mitigate potential biases in future prediction models of aneurysm rupture risk.

This study has several limitations. First, the imaging protocol determines the location of the main body of the aneurysm rather than precisely identifying the neck position relative to the DDR, which is surgically critical. Improved spatial resolution or future application of automated segmentation techniques may enhance accuracy in this regard. Second, due to the low rupture risk of most cavernous sinus-located PAs, few patients underwent surgical intervention; therefore, we could not validate our HRMRI findings against intraoperative observations, which remain the gold standard. Third, the sample size is relatively small and derived from a single center over a six-month period, which may introduce selection bias and limit generalizability. Finally, while two experienced physicians reviewed the images, formal inter-observer reliability testing (e.g., kappa statistics) was not performed. Future multicenter studies with larger cohorts and blinded multi-reader assessments are warranted to further validate this protocol.

## Conclusions

In conclusion, the HRMRI sequence may assist in determining the location of paraclinoid aneurysms, helping to distinguish whether the aneurysm is situated in the subarachnoid space or within the cavernous sinus. This information could support clinical decision-making, particularly in patients who require non-invasive, radiation-free evaluation. However, long-term follow-up remains essential even for aneurysms deemed to be located in the cavernous sinus. Further studies with surgical correlation are needed to confirm these findings. 

## Data Availability

Data are available upon reasonable request.

## Abbreviations

PAs: Paraclinoid aneurysms
HRMRI: High-resolution MRI
ICA: Internal carotid artery
Type S: Superior ophthalmic segment
Type V: Ventral ophthalmic segment
Type M: Medial clinoidal segment
Type L: Lateral clinoidal segment
Type P: Posterior clinoidal segment
CSF: Cerebrospinal fluid
IAs: Intracranial aneurysms
SAH: Subarachnoid hemorrhage
PA: Paraclinoid aneurysm
ICA: Internal carotid artery
UIAs: Unruptured aneurysms
DDR: Distal dural ring
OS: Optic strut
CSF: Cerebrospinal fluid
